# Dosimetrical and radiobiological approach to manage the dosimetric shift in the transition of dose calculation algorithm in radiation oncology: how to improve high quality treatment and avoid unexpected outcomes?

**DOI:** 10.1186/s13014-018-1005-2

**Published:** 2018-04-03

**Authors:** Abdulhamid Chaikh, Jarkko Ojala, Catherine Khamphan, Robin Garcia, Jean Yves Giraud, Juliette Thariat, Jacques Balosso

**Affiliations:** 1 Department of Radiation Oncology and Medical Physics, University Hospital of Grenoble Alpes (CHUGA), Grenoble, France; 2 France HADRON National Research Infrastructure, IPNL, Lyon, France; 3 Laboratoire de Physique Corpusculaire IN2P3/ENSICAEN - UMR6534 - Unicaen - Normandie Université, Caen, France; 4 Department of Oncology, Tampere University Hospital (Tays), Tampere, Finland; 5 Department of Medical Physics, Tampere University Hospital (Tays), Tampere, Finland; 6 Department of Medical Physics, Institut Sainte Catherine, Avignon, France; 7 Department of Radiation Oncology, Centre François Baclesse, Caen, France

**Keywords:** Equivalent uniform dose, Acuros XB, Anisotropic analytical algorithm, Radiotherapy, Dose calculation

## Abstract

**Background:**

For a given prescribed dose of radiotherapy, with the successive generations of dose calculation algorithms, more monitor units (MUs) are generally needed. This is due to the implementation of successive improvements in dose calculation: better heterogeneity correction and more accurate estimation of secondary electron transport contribution. More recently, there is the possibility to report the dose-to-medium, physically more accurate compared to the dose-to-water as the reference one. This last point is a recent concern and the main focus of this study.

**Methods:**

In this paper, we propose steps for a general analysis procedure to estimate the dosimetric alterations, and the potential clinical changes, between a reference algorithm and a new one. This includes dosimetric parameters, gamma index, radiobiology indices based on equivalent uniform dose concept and statistics with bootstrap simulation. Finally, we provide a general recommendation on the clinical use of new algorithms regarding the dose prescription or dose limits to the organs at risks.

**Results:**

The dosimetrical and radiobiological data showed a significant effect, which might exceed 5–10%, of the calculation method on the dose the distribution and clinical outcomes for lung cancer patients. Wilcoxon signed rank paired comparisons indicated that the delivered dose in MUs was significantly increased (> 2%) using more advanced dose calculation methods as compared to the reference one.

**Conclusion:**

This paper illustrates and explains the use of dosimetrical, radiobiologcal and statistical tests for dosimetric comparisons in radiotherapy. The change of dose calculation algorithm may induce a dosimetric shift, which has to be evaluated by the physicists and the oncologists. This includes the impact on tumor control and on the risk of toxicity based on normal tissue dose constraints. In fact, the alteration in dose distribution makes it hard to keep exactly the same tumor control probability along with the same normal tissue complication probability.

## Background

The main challenge in radiation therapy is to obtain the highest probability of tumor control, or cure, with the lowest amount of morbidity and toxicity to normal tissues. The continuous advances in technology provide successive generations of Treatment Planning Systems (TPS), which include more and more accurate dose calculation algorithms, able to continuously optimize the accuracy, the security and hopefully the clinical outcome of treatments. Historically, the algorithms were based on dose-to-water D(w,m) mode; all tissues are assumed to be water-like but have different density as defined in the CT density calibration curve. In the past decade, a dose-to-medium D(m,m) mode was proposed, taking into account the true tissue density, the atomic composition for each voxel, which was considered closer to the physical reality. However, the clinical use of D(m,m) has been a topic of debate for years [1–4]. In favor of D(w,m) is the fact that the clinical knowledge is based on the D(w,m), which is a simple surrogate for the cell nucleus dose in different tissues assuming nuclei compositions to be tissue independent, and that radiotherapy radiation sources are calibrated using D(w,m). In favor of D(m,m) mode is the fact that the conversion from D(m,m) to obtain D(w,m) introduces an additional uncertainty and that D(m,m) reflects the physical reality. Some differences between D(w,m) and D(m,m) could be observed in the lung, head and neck cases due to the differences between tissue densities (lung or bone) compared to water.

Considering the central role of dose calculation, the commissioning of a new advanced algorithm is a critical process to be managed with caution. Two main steps can be identified. In the first step, the medical physicists must assess the installation and configuration using national and international quality assurance (QA) protocols [5]. The second step is to measure the *dosimetric shifts* presumably introduced by the new algorithm. This should be done by using several patient treatment plans and by calculating and comparing the dose distributions obtained with both algorithms: the former one as a reference, D(w,m), and the new one, (e.g. D(m,m)). The first step is a basic duty of the medical physicist, but the second step is sometimes neglected and, actually, there are no real recommendations to manage it.

However, if the step 2 is ignored or unrecognized, overdosage or underdosage may result and thus the tumor control probability (TCP) and the normal tissue complication probability (NTCP) might be significantly modified [6]. In addition, physicists should be able to provide explanations to radiation oncologists regarding the differences of dose distributions they could observe and have to manage. This includes the dose prescription, the compliance with dose constraints and the tumor coverage by the prescribed dose, etc. The radiation oncologists should be able to make the relevant medical decision associated with this transition to get the same favorable outcomes, compared with the previous situation taken as reference.

To address this situation, based on our previous works [6, 7], we promote the application of a four-dimensional analysis to estimate the dosimetric alterations and predict the clinical changes between the reference algorithm and the new algorithm. These dimensions are: i) dosimetric, ii) global, iii) radiobiological based on the equivalent uniform dose (EUD) concept and iv) statistical. We will also provide a general recommendation about the clinical use of D(m,m) regarding the dose prescription (Dpr) or dose limits to the organs at risks (OARs), as for example in lung cancer treatment.

## Methods

### Dose calculation algorithms

The former dose calculation algorithms, types (A) and (B), such as pencil beam, convolution/superposition, analytical anisotropic algorithm (AAA), etc. are based on D(w,m) mode [8, 9]. The D(w,m) in the algorithms can be converted back to D(m,m). Conversely, the Acuros XB (AXB) algorithm, as type (C), uses the Linear Boltzmann transport equation (LBTE) providing both modes D(w,m) and D(m,m). In type (C), the absolute dose in each voxel is calculated using the determined electron angular fluence, the macroscopic electron energy deposition cross sections, and the material density of the voxel. Among these algorithms, the AXB shows the highest accuracy between measurements and dose calculations and is closer to full Monte Carlo (MC) simulations [10–14].

The D(m,m) mode is recently made available in treatment planning. Thus, the dose distribution with D(m,m) could be compared to reference one as D(w,m) where the dose limits are well established and used in radiation oncology.

### Clinical example

The following analyses from lung cancer radiotherapy data present an overview of how the QA process may be used for the evaluation of real differences between treatment plans from different dose calculation algorithms. The recent improvement of dose calculation D(m,m) vs D(w,m) was used as an example of a dose calculation model change, producing a true situation of decision in radiotherapy as well as the future transition for all radiotherapy departments seeking to improve radiotherapy plans and approaching the truest calculated DVH.

The magnitudes of dose differences depend on the type of algorithm transition and of the reference one. The methods described in this study have been applied to lung cancer with photon beams showing an example of transition (e.g, moving from pencil beam convolution (PBC) with no heterogeneity correction (PBC-NC) to modified Batho’s density correction method (PBC-MB) or moving from AAA to AXB D(m,m)).

### Quality assurance method

#### Normalization methods to compare dose calculation algorithms

To compare different dose calculation algorithms, all dosimetric data are calculated with a unique set of images for a given patient, whatever the number of different algorithms to compare.

A brief summary of the QA requirements for the process to ensure the clinical validation of a new dose calculation algorithm is the following [15]:

- a three-dimensional conformal radiotherapy (3DCRT) plan is initially generated for each chosen case to deliver, with the best possible conformation, the prescription dose (Dpr). This is the reference Plan 1. The 3DCRT is a convenient technique to evaluate the real impact of the change of a dose calculation algorithm regarding the *monitor units* (MUs) and dose distribution. This irradiation technique allows to limit the technical parameters to the minimum, conversely to more complicated Intensity-Modulated Radiation Therapy (IMRT) technique. The Plan 1 should be normalized at the isocentre (Diso) defined as corresponding closely to the center of the planning target volume (PTV). The Dpr should cover 95% of the PTV, showing a real treatment plan meeting the radiotherapy goal: maximizing dose to PTV while minimizing dose to OARs.
- the test plan, Plan 2, uses exactly the same beams as the Plan 1, recalculated for each field with the new algorithm, for *the same* Dpr as Plan 1.
- a complementary plan, Plan 3, is generated using *the same* MUs of the reference Plan with the same beam arrangements. The dose distribution of Plan 3 shows actually the dose distribution of the former treatments (Plan 1) as recalculated with the new algorithms.
- field sizes and shapes in all plans should be identical using the beam’s-eye view projection of the PTV, or Gross tumor volume (GTV).

**Fig. 1:**
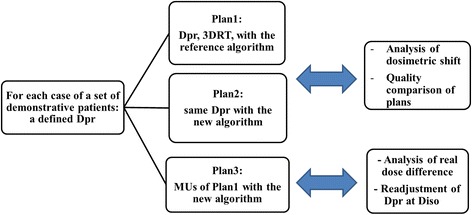
The successive design of the Plans 1, 2 and 3 for each patient case. The Plans 1 and 2 have the same Dpr but are calculated with the two algorithms to compare the reference one and the new one, respectively. Plan 3 is retro-calculated by the new algorithm with the exact amount of MUs obtained for each beam of Plan 1

There are different modes of Dpr, the most popular being either the Dpr to the isocentre (Diso) as recommended by International Commission on Radiation Units & Measurements.

(ICRU) reports 50, 62 and 83 [16–18] or setting that at least 95% of the Dpr should cover the entire PTV or that 95% of the PTV should receive at least the Dpr (D95% = Dpr), etc. Under the above conditions, the maximum dose within the target could range between about 95% and 105% of the Dpr. Any mode of dose prescription is compatible with the procedure described hereby. The Fig. 1 shows the successive generation of the Plans 1, 2 and 3 for each patient case.

### QA procedure

**Fig. 2:**
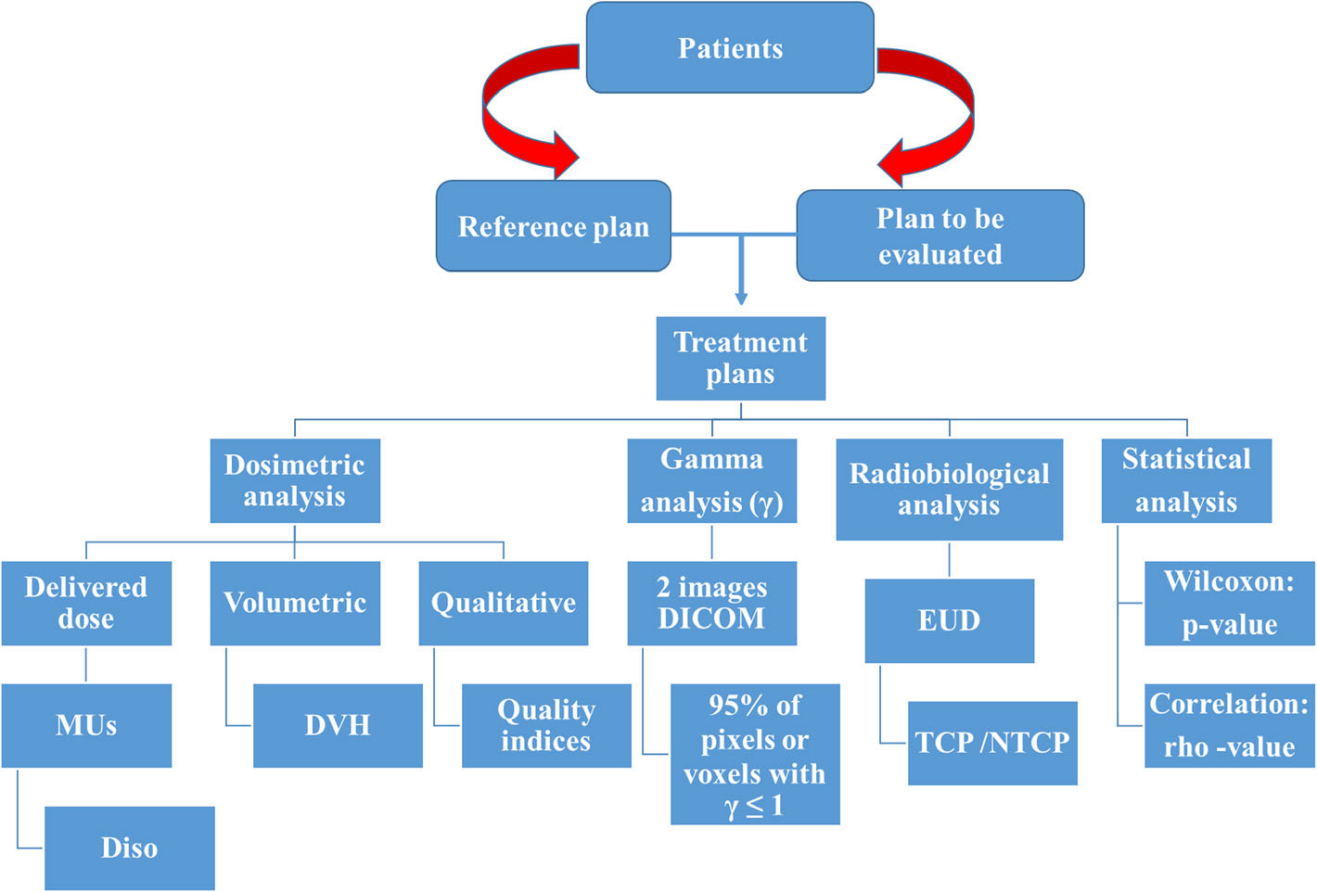
The QA method to measure and assess *the dosimetric shift* of a new dose calculation algorithm before its implementation in clinical practice, including dosimetric, global, radiobiological and statistical analysis. Abbreviations: MUs = monitor units, Diso = dose at isocentre, DVH = dose volume histograms, EUD = equivalent uniform dose, TCP = tumor control probability, NTCP = normal tissue complication probability

The Fig. 2 summarizes this QA method to measure and assess the dosimetric shift of a new dose calculation algorithm including dosimetric analysis, gamma indices (γ), radiobiological and statistical analysis.

#### Delivered dose

The MUs can be used as QA tool to compare and validate photon dose calculation algorithms. The MUs from the former/reference algorithm could be re-used to recalculate the delivered dose (DD), in Plan 3, at the reference point: Diso. The dose differences, ΔDiso, for recalculated Diso with the new algorithm depend on the magnitude of the ΔMUs (between Plans 1 and 2):

- If ΔMUs > 0, showing (MUs from ref. Plan 1 > MUs from tested Plan 2), the Diso will be higher in Plan 3 than in the reference Plan 1.
- If ΔMUs < 0, showing (MUs from ref. Plan 1 < MUs from tested Plan 2), the Diso will be lower in Plan 3 than in the reference Plan 1.

#### Dose volume histograms (DVH) indices

The QA process should be performed for each cancer site for both target and OARs. Anatomical regions with the most heterogeneous tissue densities are also the most prone to have dosimetric shifts. The DVH should be recalculated with the new dose calculation algorithms using firstly the same Dpr (Plan 2) and secondly with the same MUs from former one (Plan 3), as mentioned above. The beam arrangements, geometry and rotation should be similar in all plans without any supplementary optimization. The most important parameters are the dose near minimum (D98%), the dose near maximum (D2%, and the mean/average dose (Dmean). In addition, dose volume indices as the percent volume that received at least 95% of the prescription dose (V95%), D95%, as well as quality indices are recommended. The impact of the change would result in different DVH parameters, leading to significant impacts on quality indices. The higher/lower doses translate into overestimation or underestimation of the delivered doses and thus influencing TCP/NTCP values. The D95% for PTV should be as close as possible to the Dpr, in order to avoid the under irradiation of the tumor. On the other hand, a higher D95% would predict a higher TCP value.

The D95% could be used as indicator to readjust the Dpr and correlate with TCP values [19]:

- D95% (new algorithm) ≈ D95% (former one with ∆D95% < 2%) i.e. no adjustment is needed
- D95% (new algorithm) ≠ D95% (former one) i.e. an adjustment is to be considered

#### Gamma analysis

The γ index is a very useful tool for comparing measured and calculated dose differences, in situations where the measurement uncertainties introduce a mix of positional and dosimetric uncertainties. This tool combines two criteria including the dose difference in percentage (%) and the distance-to-agreement (DTA) in millimeters (mm). An ellipse is used to determine the acceptance region, γ ≤ 1 representing fulfillment of the criteria [20]. Since γ analysis generates a value for all points in a distribution, this value contains information about the magnitude of any disagreement in the dose and DTA from two planning algorithms. Thus, to make an overall comparison, a novel approach using 2D or 3D has been proposed. The utility of γ for comparing the results of two planning algorithms has been demonstrated by several works [21, 22]. For γ analysis, the Digital Imaging and COmmunications in Medicine (DICOM) data including dose distribution from reference and tested algorithms for each patient should be exported from TPS. The results per treatment plan could be calculated by considering all pixels for a specific patient using axial, sagittal and coronal plans. The results are displayed using a γ -maps and cumulative Pixels-γ-Histogram (PγH).

The γ-maps show the pixels with γ > 1 that were out of tolerance, indicating overestimated or under estimated doses. We could then discriminate the healthy tissues located around the target volumes. The superposition of the γ-map with the computed tomography (CT scan) provides the anatomical information, showing in color, where the dose differences are located helping the radiation oncologist for decision-making. The PγH indicates the fraction of pixels with a γ-indices ≤1. We considered that dose distributions from both algorithms were similar, if 95% of pixels or voxels are passing the γ-criteria with γ ≤ 1.

It is interesting to note that, there are also some other techniques to compare dose distributions more or less similar to γ, such as delta envelope. However, caution should be done when comparing dose distribution from former algorithm with dose distribution with MC to avoid the overestimated or underestimated average γ-value or γ-passing rate due to the increase of the statistical noise level in the dose distributions computed with MC simulation [23–25].

#### Radiobiological analysis

The DVH for both target and OARs could be used to determine respectively the TCP and NTCP from a treatment plan with a specific Dpr. The most important parameter that correlates with the TCP is the Dpr translated by the TPS into DD with MUs. However, when changing a dose calculation algorithm, the dose distribution will change and it would be hard to get exactly the same TCP and NTCP values, compared to the reference one. In this context, to correlate the real DD with Dpr, the EUD concept was shown to be a useful indicator to compare the dose distribution, coming from different algorithms, for the target volume and OARs [26].

According to Niemierko’s model, EUD is defined as [27, 28]:

1 $$ EUD={\left(\sum \limits_i{v}_i{D}_i^a\right)}^{1/a} $$

where (v_i_) is the fractional organ volume receiving a dose (D_i_) and (a) is a tissue specific parameter, easy to find in the literature, that describes the volume effect. It is one of the problems of EUD’s applicability, that tissue specific parameters, such as (a) are not readily described.

The TCP and NTCP could be calculated as:

2 $$ TCP=\frac{1}{1+{\left(\frac{TCD_{50}}{EUD}\right)}^{4{\gamma}_{50}}} $$

3 $$ NTCP=\frac{1}{1+{\left(\frac{TD_{50}}{EUD}\right)}^{4{\gamma}_{50}}} $$

where TCD_50_ is the dose to control 50% of the tumors when the tumors are homogeneously irradiated. TD_50_ is the tolerance dose for 50% complication rate of the normal organ. The factor (γ_50_) describes the slope of the dose-response curve.

As shown in eq. 1, the EUD concept combines dose distribution with a radiobiological parameter (a), and reflects the biological properties of the tumors and organs. The parameter (a) has a negative value for tumors, and a ≥ 1 for OARs. The values (a = 1/n) for OARs can be taken from Lyman-Kutcher-Burman (LKB) model [29, 30].

By definition, D98% < EUD < D2%:

- when a < 1, for target volumes (e.g a = − 10 for the lung), the model weights more on the low dose area and EUD becomes D98%;
- when a = 1, for parallel organs that exhibits a large volume effect as lung, the EUD becomes Dmean and thus NTCP value depends on Dmean;
- when a > 1, for serial organs such as the spinal cord, the model weights more on the high dose area to penalize hot spots and EUD becomes close to D2%.

If the parameter (a) cannot be calibrated for the calculation of EUD, a confidence interval around the calculated EUD values by calculating the lower and upper bounds on the EUD can be estimated, using a = (0.5–3.0) for parallel organs, and a = (4.0–15.0) for serial organs [31].

To obtain TCP or NTCP equal to 50%, which is the most sensitive part of the sigmoid dose-response curves, the TCD_50_ or TD_50_, respectively, should be equal to the EUD values derived from DVH. To avoid the uncertainties associated with the use of TCP and NTCP running with obsolete radiobiological parameters, Chaikh et al. 2016, proposed to use the EUD concept to validate the new dose calculation algorithms in a radiobiological perspective. Consequently, the EUD resulting from a given treatment, taken as reference, could be the gold standard to obtain the desired TCP or NTCP values, since they depend on EUDs. In addition, it could be used as an objective for optimization [26].

As a whole, if the new algorithms provide a lower EUD to the target, this will indicate that the target will be under irradiated compared to the reference one. This might produce unexpected recurrences. Since the expected local control is associated with the Dpr, the EUD value of the target provides essential information about the real delivered dose that should be very close to Dpr. On the other hand, the EUD for an OAR should be much lower indeed than TD_50_, as 50% of severe complications is usually not acceptable.

#### Statistical methods

As the same CT scan, for each patient, is used to generate the different treatment plans and that the dose is recalculated with the new algorithm, there is a relationship between the dosimetric data from reference plan and the tested plans with new algorithms, excluding any anatomical variation. Thus, Wilcoxon signed rank test can be used and is able to calculate a reliable *p*-value with a very small number of cases. In addition, the statistical correlation between the data could be evaluated using Spearman’s correlation coefficient (ρ-value). More recently Chaikh et al. 2016, proposed the bootstrap simulation method to estimate the minimal number of cases to observe a significant difference with *p* < 0.05. The method uses randomly chosen sample (n), iteratively drawn with replacement from the original data set accounting a cases number (m). For every n, the mean *p*-value across the 1000 random samples could be computed using Wilcoxon signed-rank test. Then the *p*-values as a function of each (n) could be plotted up to number (n = m) showing the variation of *p*-value with (n) [32].

#### Medical decision: suggested Dpr adjustments

**Fig. 3:**
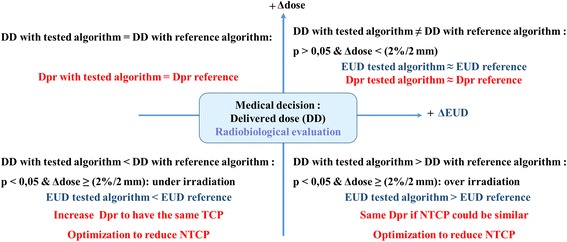
Suggested medical decisions concerning the modification of prescribed dose (Dpr) when changing the dose calculation algorithm for a new one

The final objective is to propose an approach, already tested in our department for lung radiotherapy, to check if the Dpr should be readjusted, or not, when changing the dose calculation algorithm. Considering that, if there is a statistically significant difference in dose calculation, with *p* < 0.05, the Dpr could be readjusted. The objective is mainly to keep unchanged NTCP-value. The significant difference means, with a 95% of confidence, an existing difference between former and newer algorithms. To support the medical decision, a quantitative evaluation could be carried out using dosimetric, 2D or 3D global analysis based on γ-criteria and radiobiological based on EUD concept. The Fig. 3 shows a suggested principle of medical decision concerning the modification of Dpr when moving toward a new dose calculation algorithm.

## Results

### Delivered dose

**Fig. 4:**
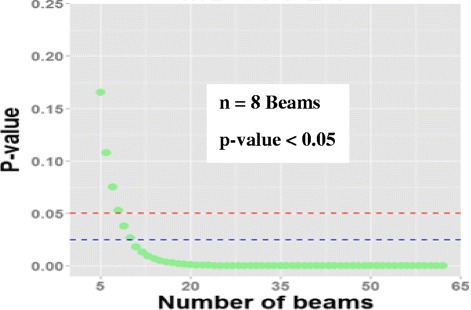
*p*-values estimated by bootstrap simulation, indicating the average *p*-value for each sample-sizes going from *n* = 5 to *n* = 62 beams. The red and the blue dashed lines corresponds to a significance threshold of 0.05 and 0.025, respectively

The aim was to compare the DD in MUs resulting from different dose calculation methods keeping exactly the same beam setting. The bootstrap analysis showed that 8–10 beams are sufficient to confirm the significant difference when moving from PBC-NC to PBC-MB or PBC-MB to AAA, and AAA to AXB D(m,m). In addition, the difference in re-evaluated Diso agrees inversely but with the same magnitude of ΔMUs for results presented in this study, as mentioned above. However, attention should be paid to MUs, since the difference in MUs depends on algorithm type as well as the transition (eg, transition from PBC-MB directly to AXB with D(m,m) mode or transition from former algorithms with D(w,m) mode to MC). Thus, the integration of MC method in clinical use needs more caution depending on reference algorithm to avoid the overirradiate or under irradiate the patient. Figure 4 shows *p*-values estimated by bootstrap simulation, indicating the average *p*-value for each sample-sizes going from *n* = 5 to *n* = 62 beams.

### Dose volume histograms indices

**Fig. 5:**
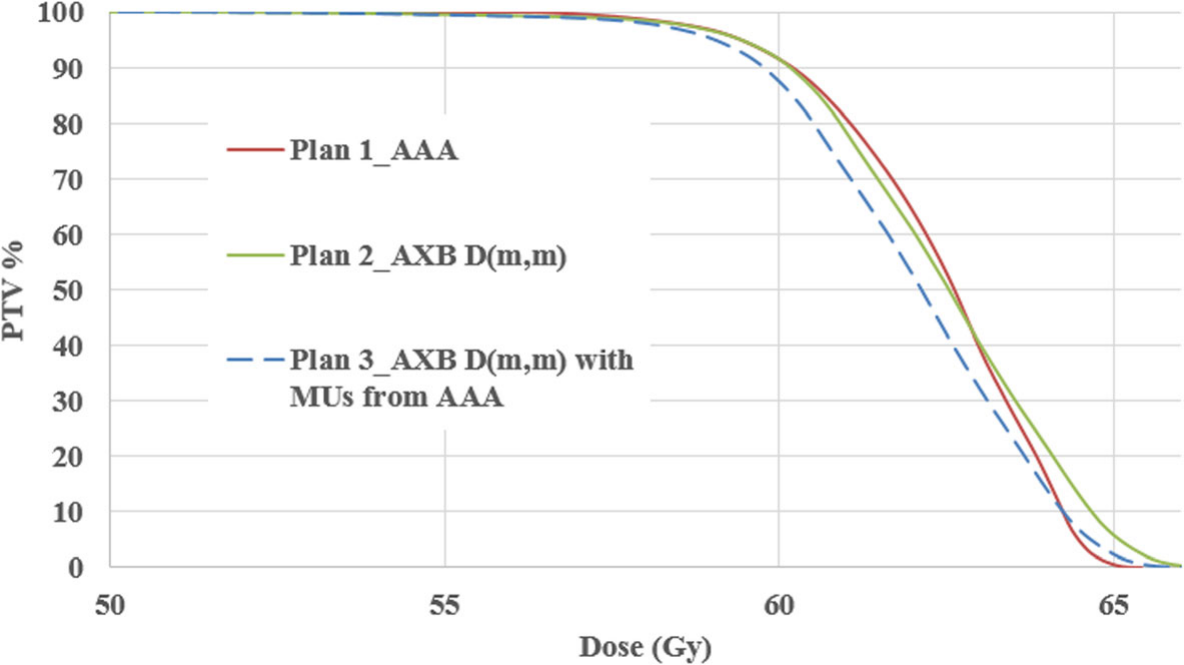
The DVH is a sensitive representation of the dosimetric shift that could also be seen on the 2D or 3D anatomical representation of dose distribution on the CT scan of the patient. The latter being the base of the γ-index analysis. Plans 1 and 2 are calculated for lung with 3DCRT using the same Dpr with two different algorithms, and Plan 3 is a retro-calculation by the new algorithm with the MUs of Plan 1

A very significant difference was observed when moving from PBC-NC to PBC-MB or from PBC-MB to AAA. It can be seen in the example presented in Fig. 5 that D95% was lower than initially calculated with reference algorithm as AAA.

### Gamma analysis

**Fig. 6:**
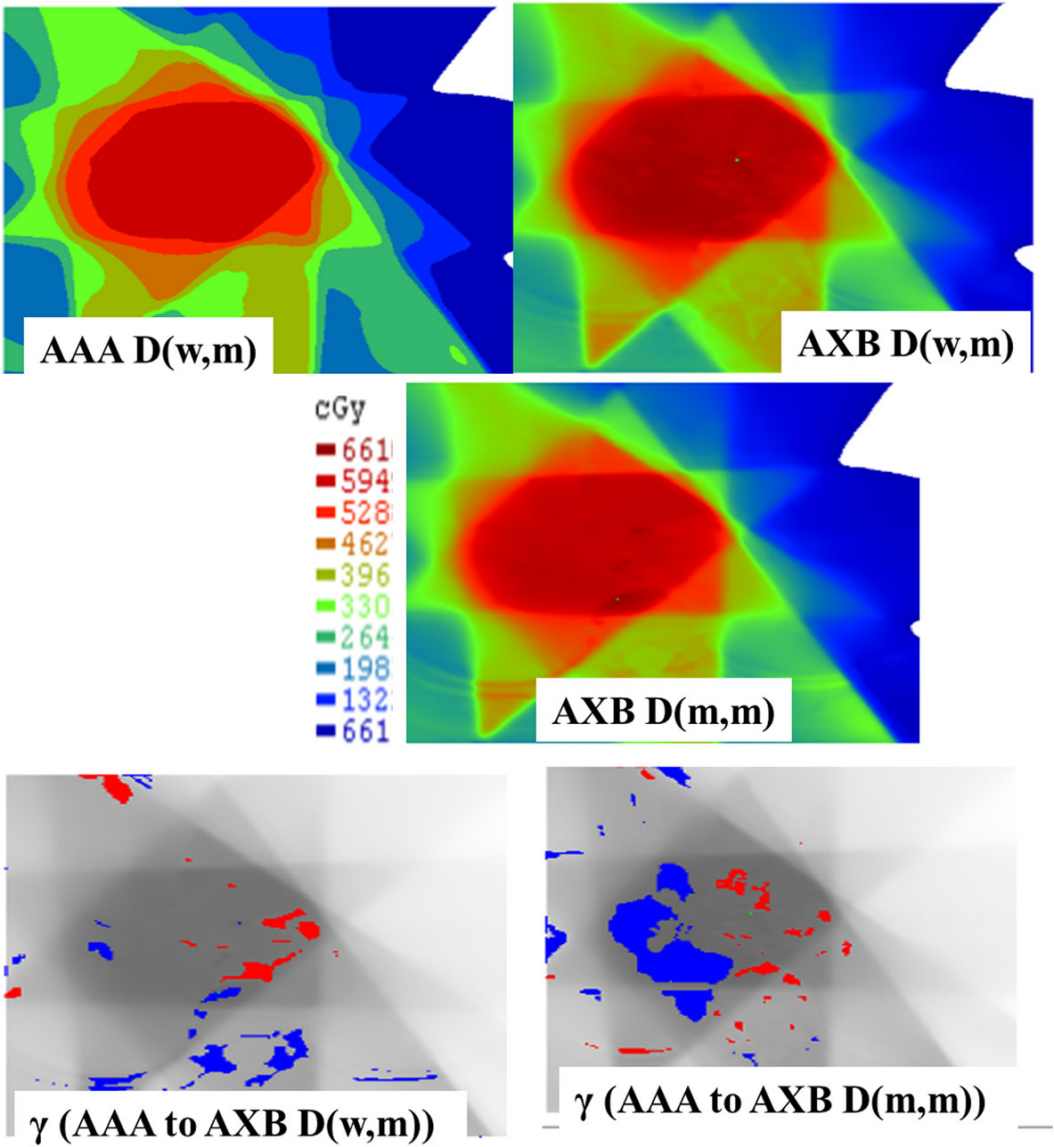
2D-γ maps plotted on the axial views for comparing AAA as D(w,m) to AXB with D(w,m) mode or AAA to AXB with D(m,m) mode. The three fully colored panels are the dose distributions respectively with the three different algorithms. The two grey backgrounded panels with the red and blue coloring in the lower part indicate pixels having γ > 1 and identify respectively overestimating and under estimating dose. In the present case the γ passing rates, 95% of pixels with γ ≤ 1, was satisfied using 2%/ 2 mm and 3%/ 3 mm respectively when moving from AAA to AXB with D(w,m) mode or AAA to AXB with D(m,m) mode. It can be seen also that the dose is more heterogeneous using both AXB modes, which might influence the TCP and NTCP values

Figure 6 shows an example of 2D γ-maps plotted on the axial views for comparing AAA to AXB with D(w,m) mode or AAA to AXB with D(m,m) mode.

### Radiobiological analysis

The analysis of EUD values showed that EUD for target from former algorithm such as PBC-MB was overestimated leading to EUD ≈ Dpr, while the more modern AXB using the same MUs showed that EUD < Dpr. This explains that in reality without heterogeneity correction for a decade the truth DD was overestimated (under irradiation of the patient). Using the more modern model with the same Dpr would lead to over irradiation of the patient. This means that keeping of same Dpr to obtain more TCP-value can only be justified if a NTCP reduction is obtained as a compensation, to maintain the lowest reasonably achievable risk. The radiobiological analysis TCP/NTCP confirmed the results from DVH parameters as higher values of D95%, Dmean and D98% with PBC-NC or PBC-MB compared to AAA or AXB.

In Table 1 an example of the use of EUD concept for lung cancer shows recommended EUD values in Gy, corresponding to absolute value of NTCP = 1% or 5% of toxicity for OARs. The recommended EUD values could be easily correlated with dose limits for OARs. The recommended dose limits could be used for all transition types to avoid the high NTCP values. However, a caution should be done regarding TD_50_ for healthy lung, since TD_50_ depends on algorithm types or model as pencil kernel or point kernel (e.g, PBC, CS, EPL, etc.). In addition, this value depends on irradiation site either from breast cancer or lung cancer. The values of TD_50_ and a = 1/n were taken from LKB parameters for tissue complications after treatments with conventional fractionation. By complying with these EUD values, in Table 1, the patients should really benefit from the use of the advanced algorithms. It is worth to remind that the dose limits depend on the type of structure. Serial organs as the spinal cord are dependent of D2%, whereas parallel organs as lung are dependent of Dmean and the volume fraction receiving a specific dose (Vdose). Thus, the recommended EUD could be matched with the dose tolerance of each organ to respect the dose limits for high quality treatment plan when integrating a new dose calculation algorithm as type (C) generation.

**Table 1:** Planning objectives and dose limits for OARs associated with lung treatment when using a new dose calculation algorithm

Structure	TCD_50_ (Gy)	a	Endpoint	EUDmin (Gy)		Dose limits
Target	50 [35]	−10	Control	EUD = Dpr TCP > 50%		D95% = Dpr V95% > 95% Dpr
OARs	TD_50_ (Gy)	a [29, 36]	Endpoint	EUDmax (Gy) NTCP ≤1%	EUDmax (Gy) NTCP ≤5%	Dose limits [37, 38] (in 3DCRT)
Lungs	24.5 [30]	1.2	Pneumonitis	13.9	17.1	Dmean< 15–20 Gy
Lungs	30.8* [39]	1.01	Pneumonitis	17.4	21.4	
Esophagus	68 [30]	18	Perforation	51.1	56.5	Dmean < 34 Gy D2% < 69 Gy
Esophagus	51* [40]	2.27	Acute esophagitis Grade 2–3	38.4	42.4	
Heart	48 [30]	3.1	Pericarditis	32.8	37.5	Dmean < 26 Gy D2% < 30 Gy
Spinal cord	66.5 [30]	20	Necrosis	49.9	< 55.3	D2% < 50 Gy

The EUD values are corresponding to clinical objectives of NTCP ≤1% or 5%. The TCD_50_ was taken from Okunieff et al. The parameters TD_50_ and a = 1/n for tissue complications with conventional fractionation were taken from Emami or more recent publications. The symbol (*) indicates the most recent and recommended tolerance dose TD_50_ for esophagus; and lung with heterogeneity correction using AAA. It can be seen that dose limits as Dmean and D2% depending on OARs (serial or parallel) could be matched with EUD values and the respect of the proposed dose limits might produce NTCP ≤5%. N.B. the Dmean = 20 Gy for healthy lung leads to a NTCP ≈ 15%

### Medical decision

The mean comparison using statistical tests indicated significant differences in dose calculation: PBC-MB vs PBC-NC or AAA vs PBC-MB and AXB with D(m,m) vs AAA. In other words, the observed differences probably reflect existing differences between the dose calculation models. In addition, the bootstrapping procedure for lung indicated that significant differences between the reference and the new methods could be observed with as little as 8 cases, since the difference in ΔMUs > 0, for all beams going in the same direction (eg, MU_AXB_ > MU_AAA_). Therefore, the difference in MUs impacted the dosimetric data showing a real clinical impact. The most advanced dose calculation algorithms, as AXB, calculated lower D98% and more dose heterogeneity inside the target compared to type (A) algorithms that may increase the probability of recurrence.

In radiobiological analysis, the magnitude of ΔEUD depends on transition type and anatomical location site and density. Thus, to ensure a reasonably low loss of TCP and/or increase in toxicity comparing to the reference algorithm, a reasonable goal would be to give at least the same Dpr; although it is rather obvious to suggest an adjustment of Dpr for each transition since Diso would be changed. However, attention should be paid to transition types, (e.g., when comparing type (A) algorithms such as PBC to type (B) algorithms, using the same Dpr, we observed that PBC overestimates DVH, and consequently the EUD. In this specific context, the EUD of plan 3 (as defined above) must be calculated with the same MUs from older algorithm to show the “real”, at least more realistic, EUD.

In this specific comparison for lung cancer radiotherapy with 3DCRT using Dpr = 60 Gy, the EUD ≈ 60 Gy calculated with PBC-MB was on average EUD = 55 Gy with the AAA using the same MUs from PBC-MB as reference one [19].

In addition, when comparing AXB vs AAA using Dpr = 60 Gy, EUD ≈ 60 Gy calculated with AAA was on average EUD = 58 Gy with the AXB D(m,m) using the same MUs from AAA as reference one. Thus, a recommendation to readjust the Dpr could be suggested. This finding agrees with international recommendations indicating that a decrease of Dpr should be suggested [33, 34]. Therefore, a reduction of Dpr from 5 to 10% is recommended when moving from type (A) algorithms to type (B) algorithms. In the same line, there is a need to readjust the dose constraints for future algorithms that use heterogeneity corrections.

## Discussion

The challenges of implementation of a new dose calculation algorithm should not be seen as a reason not to implement these algorithms, since the most accurate dose calculation algorithm would be used for lung cancer radiotherapy. In this context, there is not a guideline as to whether dose-to-water or dose-to-medium should be used. This paper presents a very useful methodology for individual departments to transition from one dose calculation algorithm to another, and also from dose-to-water to dose-to-medium. The recommendation about the choice of dose-to-medium vs dose-to water in clinical use needs more additional evidence.

Also, with auto-planning becoming more widely available, we suggest to use automation mode to compare the different algorithms and this could be made much easier. The choice of how plans are chosen and technique should be also carefully made.

Among the numerous photon dose calculation models available in TPS, the medical physicist has to make a clinical comparison well adapted to each technical transition and cancer sites treated with radiation therapy. At first, the advance in dose calculation models in radiotherapy and their principle to calculate the dose need a deep and robust analysis. In particular, one should assess whether the new dosimetric data are in the same magnitude as former data and fulfill the proposed tolerance limits (e.g, 2%/ 2 mm).

As the first step, the comparison of MUs proved to be useful for comparison of algorithms using 3DCRT. However, the comparison of two different techniques is not possible by only comparing MUs since other parameters change besides the calculation method as invers planning method and the beam weights, etc. For this reason, the 2D or 3D gamma analysis methods are convenient to compare techniques (i.e. IMRT or VMAT vs 3DCRT) since they just require DICOM files of dose distributions.

When the dosimetric data fulfill these limits, one can use these algorithms without the need to readapt the Dpr. But, if the dosimetric data do not fulfill these tolerance limits, a radiobiological evaluation should be also used, as for instance EUD concept. Moreover, attention about the calculated doses to OARs, we recommend to compare γ-maps with dose distribution to check if the tolerance dose for each organ was respected. In addition, a useful statistical tool was proposed, bootstrap, when dealing with repeated calculations. These features make the radiobiological and statistical methods particularly adapted for radiotherapy data analysis. The dosimetric data from radiotherapy plans are “statistically” paired and strong correlation would be observed, since only one parameter should be changed (dose calculation method). However, an in-depth discussion between medical physicists and radiation oncologists is strongly encouraged and recommended when implementing the new dose calculation algorithm in order to determine which radiobiological parameters are the most appropriate for this type of transition (e.g., use of TD_50_ for NTCP with AXB vs AAA or CS vs EPL). Ideally, the TD_50_ and TCD_50_ should be proposed by the real clinical outcomes from the used department.

The example given in this study concerning the Dpr is a challenge of implementation of a new dose calculation algorithm in radiotherapy. The clinical effect in radiotherapy depends on the DD, a small difference in DD should be considered for both target and OARs. Concerning the DD, we observed a significant difference when moving from PBC-NC to PBC-MB as well as type (A) to (B) or type (B) to (C) transition. The TCP and NTCP radiobiological parameters have been proposed to the previous algorithms, thus the use of their former parameters to compare more advanced algorithms will introduce uncertainties in real TCP and NTCP values. The use of a new cohort of patients treated with more advanced dose calculations is a first and necessary step to find the true dose-response relationship. On the other hand, the improvement of the estimation of TCP/NTCP requires a regular adjustment of the radiobiological parameters for each transition.

## Conclusion

This paper shows an approach to assess the so called *dosimetric shifts*: the alterations and dose differences when changing the calculation algorithm in radiotherapy. The differences between former and new algorithms depend on transition type and might exceed 5–10%. Therefore, the alterations for Diso and MUs should be assessed and taken into account in the process of QA in radiation oncology. As, the changes in ΔDiso are not all going in the same direction, this could be a source of misunderstanding between the radiation oncologists and medical physicists. These alterations could be a reduction of the delivered dose according to the type of the new algorithm. When attention is pointed on the TCP and NTCP predictions, it is difficult to find the more relevant radiobiological parameters (γ_50_, TD_50_ and TCD_50)_ in the literature. Therefore, the EUD can be used to avoid the over prediction or under prediction related to these uncertainties of radiobiological parameters. Ideally, each radiation oncology department should be able to assess this change using an approach comparable to the one described here to build a valuable medical decision with at least a small set of patients using bootstrap simulation.

## Abbreviations

3DCRT: Three-dimensional conformal radiotherapy
AAA: Analytical anisotropic algorithm
CT: Computed tomography, and a: tissue specific parameter
D(m,m): Dose-to-medium
D(w,m): Dose-to-water mode
D2%: The dose near maximum
D95%: The minimum coverage dose of 95% of the target
D98%: Dose near minimum
DD: Delivered dose
DICOM: Digital Imaging and COmmunications in Medicine
Diso: Dose at the isocentre
Dmean: The mean dose
Dpr: Dose prescription
DTA: Distance-to-agreement
DVH: Dose volume histograms
EUD: Equivalent uniform dose
GTV: Gross tumor volume
ICRU: International Commission on Radiation Units & Measurements
LBTE: Linear Boltzmann transport equation
LKB: Lyman-Kutcher-Burman
MC: Monte carlo
MUs: Monitor units
NTCP: Normal tissue complication probability
OARs: Organs at risks
PBC: Pencil beam convolution
PBC-NC: PBC with no heterogeneity correction, modified Batho’s density correction method (PBC-MB)
PTV: Planning target volume
PγH: Pixels-γ-Histogram
QA: Quality assurance
TCD_50_: The dose to control 50% of the tumors when the tumors are homogeneously irradiated
TCP: Tumor control probability (TCP)
TD_50_: The tolerance dose for 50% complication rate of the normal organ
TPS: Treatment Planning Systems
V95%: The percent volume that received at least 95% of the prescription dose
γ: Gamma index
γ_50_: The slope of the dose-response curve
